# Age-related changes in skeletal muscle: changes to life-style as a therapy

**DOI:** 10.1007/s10522-018-9775-3

**Published:** 2018-09-27

**Authors:** Rachel McCormick, Aphrodite Vasilaki

**Affiliations:** Musculoskeletal Biology II, Institute of Ageing and Chronic Disease, Centre for Integrated Research into Musculoskeletal Ageing, University of Liverpool, William Duncan Building, 6 West Derby Street, Liverpool, L7 8TX UK

**Keywords:** Sarcopenia, Skeletal muscle ageing, Muscle wasting, Sarcopenic therapeutics

## Abstract

As we age, there is an age-related loss in skeletal muscle mass and strength, known as sarcopenia. Sarcopenia results in a decrease in mobility and independence, as well as an increase in the risk of other morbidities and mortality. Sarcopenia is therefore a major socio-economical problem. The mechanisms behind sarcopenia are unclear and it is likely that it is a multifactorial condition with changes in numerous important mechanisms all contributing to the structural and functional deterioration. Here, we review the major proposed changes which occur in skeletal muscle during ageing and highlight evidence for changes in physical activity and nutrition as therapeutic approaches to combat age-related skeletal muscle wasting.

## Skeletal muscle ageing

Sarcopenia is defined as the loss of muscle mass and function as we age (Rosenberg 1989). In humans, sarcopenia affects individuals from approximately the 4th decade of life (Lexell et al. 1988), with a decrease of 30–50% in skeletal muscle mass and function by the time individuals reach approximately 80 years of age (Akima et al. 2001) and this is worsened by unloading of muscle in inactive old people (Bamman et al. 1998; Breen et al. 2013).

**Fig. 1:**
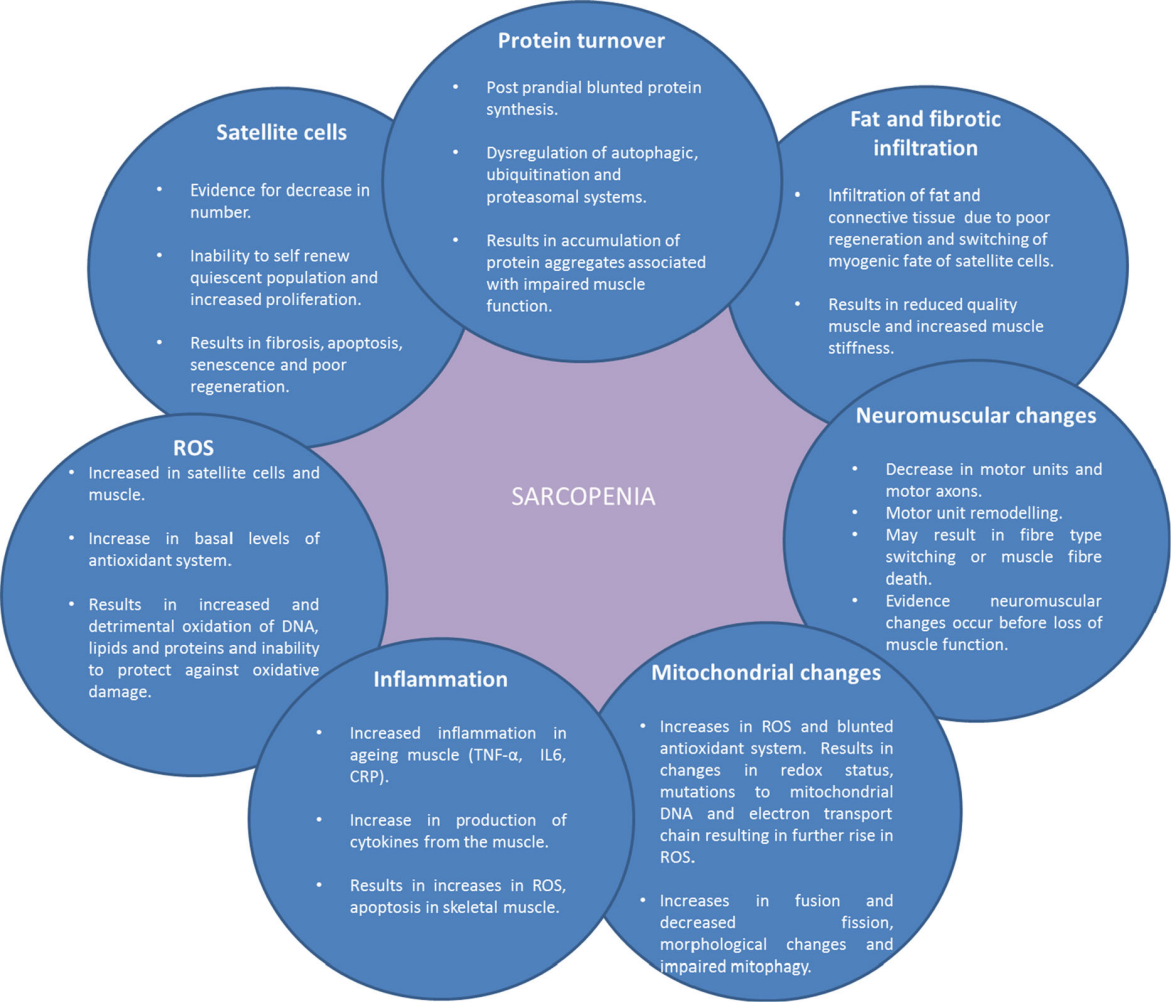
Summary of changes that occur within the aged skeletal muscle and the role they play in sarcopenia

The mechanisms that underlie sarcopenia are not completely understood and it is likely that it is a multifactorial condition with a network of interacting dysfunctional systems (Fig. 1). Among several proposed processes are: decrease in protein synthesis (Welle et al. 1993), infiltration of fat tissue and connective tissue into skeletal muscle (Brack et al. 2007; Addison et al. 2014), dysregulation of proteasomal degradation pathways (Chondrogianni et al. 2000; Cuervo and Dice 2000), mitochondrial dysfunction (Short et al. 2005; Sakellariou et al. 2013), reduced number of satellite cells (Shefer et al. 2006), increased ROS production (Broome et al. 2006; Palomero et al. 2013) and increased inflammation (Fagiolo et al. 1993). These processes are proposed to lead to a decrease in muscle fibre number, decreased muscle cross-sectional area and defective regeneration observed in older humans (Lexell et al. 1988; Carlson et al. 2001). Fibre type changes have been proposed to be one of the important mechanisms of loss of muscle function with ageing, with type II fibres being more susceptible than type I fibres to atrophy (Larsson et al. 1978; Lexell et al. 1988; Nilwik et al. 2013). There is also evidence for increases in the ratio of type I to type II fibres in humans (Larsson et al. 1978; Larsson 1995; Andersen 2003; Lee et al. 2006). However, conflicting data exists demonstrating no difference in the percentage of type I and type II fibres with age in humans (Lexell et al. 1988). The reasons for these contradicting findings are unclear however it may be due to the age range of participants in Lexell’s study being slightly younger than the other studies. Furthermore, the demographics and lifestyles of the participants are not stated in the majority of the studies, therefore it is possible this may affect results. For example, the level of activity may have been lower in the younger individuals, and higher in the older participants than those in other studies in Lexell’s study.

## Changes in satellite cells during sarcopenia

Satellite cells are stem cells present in adult muscle and are necessary for skeletal muscle to regenerate following injury (Shafiq and Gorycki 1965; Fry et al. 2015). During ageing, the number of satellite cells have been shown to decline in mice (Day et al. 2010; Chakkalakal et al. 2012) in a fibre type specific manner; in humans a decrease in satellite cells in type II fibres, with no differences seen in type I fibres have been shown in humans (Verdijk et al. 2014). Furthermore, a decline in the satellite cell content of *extensor digitorum longus* (EDL) muscle (primarily type II fibre) of mice is seen at 1 year of age, whereas this decline was not seen in the soleus muscle (predominately type I fibre) until mice were 2.5 years old (Shefer et al. 2006). Remarkably, this study also showed the presence of whole myofibres with no satellite cells present in 2.5 year old mice (Shefer et al. 2006). However, a study by Carlson et al. (2001) showed that satellite cell number was increased in muscles of old rats suffering from hind-limb neuropathy (Carlson et al. 2001). These studies used different species as well as different muscles and suggest that changes in satellite cell number may be specific to the muscle and species used. Ballak et al. showed that proteins involved in satellite cell proliferation from mice were not grossly affected during ageing (Ballak et al. 2015), however in a study by Shefer et al. the initial proliferation rate of isolated satellite cells from old mice in culture was lower than in cells isolated from younger mice (Shefer et al. 2006).

Satellite cells normally self-renew the quiescent pool of satellite cells (Zammit et al. 2004). During ageing the ability of satellite cells to self-renew is reduced (Shefer et al. 2006) due to the increase in proliferation (Chakkalakal et al. 2012) which can lead to apoptosis or senescence (Sousa-Victor et al. 2014). This may contribute to sarcopenia development and is associated with poor regeneration of muscle of aged animals (Carlson et al. 2001). Furthermore, the loss of satellite cells has been associated with neuromuscular degeneration during ageing (Liu et al. 2017).

The role of altered properties of satellite cells as an underlying cause of sarcopenia is unclear, as depletion of satellite cells from muscles of old mice had no effect on the cross-sectional area of the muscle (Fry et al. 2015) or on muscle growth after unloading (Jackson et al. 2012). Moreover, parabiosis studies showed that muscle of old mice can regenerate successfully when placed in a young host (Carlson and Faulkner 1989; Conboy et al. 2005). In vitro studies have also shown that satellite cells isolated from old mice can differentiate into mature myotubes (Shefer et al. 2006) and when supplemented with fibroblast growth factor (FGF), no difference was shown in the ability of satellite cells from adult and old mice to proliferate ex vivo. These data suggest that the changes in the satellite cell environment, rather than loss of function within satellite cells, during ageing are likely to cause the dysfunction of satellite cells (Shefe*r* et al. 2006; Lee et al. 2013). However, the ablation of satellite cells results in increased fibrosis suggesting satellite cell function may play a role in preventing fibrosis. Further studies have also shown that satellite cells are essential for regeneration following damage in muscle since ablation of these cells had profound effects on the ability of muscles to successfully regenerate in mice (Fry et al. 2015).

Some of the major pathways associated with these changes in satellite cells during ageing include Notch and Wnt signalling. Notch signalling is associated with the proliferation of satellite cells whereas canonical Wnt signalling is associated with the differentiation of muscle cells (Brack et al. 2007), however the involvement of Wnt in muscle differentiation is debated (Murphy Malea et al. 2014). During ageing there is a decrease in Notch signalling (Carey et al. 2007) and a switch from the canonical Wnt signalling to non-canonical Wnt signalling, this results in the prevention of the self-renewal ability of satellite cells that is seen in the aged satellite cell (Florian et al. 2013). Key genes which regulate skeletal muscle development, MyoD and Myf5 are also increased in the aged muscle in humans, mouse and rats (Hameed et al. 2003; Raue et al. 2006; Chakkalakal et al. 2012).

## Changes in protein synthesis during sarcopenia

A balance between protein synthesis and degradation is vital to maintain muscle mass and the relevant gains or losses in protein synthesis and degradation rates are required for hypertrophy and atrophy. Studies of basal levels of protein synthesis have shown contradicting results with some studies demonstrating decreased rate of overall protein synthesis in the muscle of old compared with adult humans (Hasten et al. 2000) and other studies showing no difference in protein synthesis in muscles of old humans compared with muscles of adults (Volpi et al. 2001; Wall et al. 2015; Francaux et al. 2016). Thus, there is a lack of consistent evidence for differences in basal protein synthesis between young and old people. Therefore, research has focused on studying post-prandial state protein synthesis to identify whether older people can utilise protein as efficiently as younger people. These studies have shown that older people have a blunted protein synthesis response to nutrients (Cuthbertson et al. 2005; Wall et al. 2015) and to exercise (Fry et al. 2011); this is known as anabolic resistance. Data by Koopman et al. (2009) demonstrated that there was no difference in either digestion or absorption of proteins between the old and young people therefore anabolic resistance was proposed to result from an increase in the amount of protein required to reach a ‘threshold’ for protein synthesis to occur (Koopman et al. 2009). This is further evidenced by studies that show blunted mTOR activation following protein intake in the muscle of older people (Cuthbertson et al. 2005). Although anabolic resistance is likely to contribute to the onset of sarcopenia, it is unlikely that it contributes to the continuous decrement in muscle mass seen in sarcopenia as an increase in anabolic resistance did not have any detrimental effect on muscle mass (Smeuninx et al. 2017).

## Changes in protein degradation during sarcopenia

The appropriate quality control of protein is vital for the correct functioning of the cell. Two common mechanisms responsible for this are the proteasomal degradation pathway and autophagy. These two pathways are dysregulated in a host of tissues during ageing and therefore are hypothesised to contribute to the loss of muscle mass with age.

## Proteasomal degradation during sarcopenia

The role of the ubiquitin–proteasome system (UPS) is to regulate protein degradation and maintain protein homeostasis. Proteins are labelled with ubiquitin molecules for degradation and are passed to the proteasome where they are degraded.

There are numerous ligases able to carry out protein degradation; however Atrogin-1 and muscle RING-finger protein-1 (Murf1) are muscle specific ligases that play a role in numerous models of muscle atrophy (Bodine et al. 2001). Despite the evidence for a role of the UPS in muscle atrophy, the role of the UPS in sarcopenia is controversial. Some studies have shown the upregulation of both Atrogin-1 and Murf1 levels in the muscle of old rats (Clavel et al. 2006), whilst others have shown no difference or downregulation between the age groups (Gaugler et al. 2011) or the upregulation of only one of the atrogenes (Altun et al. 2010). The contrasting results in these studies maybe due to the transient nature of these two atrogenes making it difficult to accurately identity changes in their expression levels (Bodine et al. 2001; Sacheck et al. 2007).

## Autophagy during sarcopenia

Autophagy is the process of “self-eating” and is crucial for the turnover of cell components, both in normal circumstances as well as during cellular stress such as starvation (Pfeifer and Warmuth-Metz 1983). As opposed to the UPS which is only able to degrade proteins, the lysosomal system is able to incorporate protein aggregates, macromolecules and whole organelles (Korovila et al. 2017). Reduced autophagy has been seen in many cell types and tissues during ageing (Cuervo and Dice 2000; Kiffin et al. 2007) and there is evidence that autophagy is dysregulated in the muscle of old rodents (Russ et al. 2012; Joseph et al. 2013b; Russ et al. 2015a). Studies in *Drosophila* have shown accumulation of protein aggregates in muscle that was associated with impaired muscle function (Demontis and Perrimon 2010), providing evidence for autophagic dysregulation in the development of sarcopenia.

Impairment of mitophagy (autophagy of the mitochondria) is detrimental to muscle homeostasis, and leads to the accumulation of damaged and dysfunctional mitochondria (Grumati et al. 2010). Dysfunctional mitophagy has been shown to occur in the muscle of old men (Gouspillou et al. 2014) and women (Drummond et al. 2014) and is therefore hypothesised to play a role in the mitochondrial dysfunction seen in sarcopenia.

## Infiltration of fat and fibrosis during sarcopenia

Fibrosis is the accumulation of extracellular matrix (Alnaqeeb et al. 1984; Goldspink et al. 1994) and during sarcopenia both fibrosis and the infiltration of fat into skeletal muscle occurs (Evans et al. 1995; Song et al. 2004). This decrease in the quality of skeletal muscle is thought to contribute to the age-related impairment in force generation, particularly in lateral transfer of force throughout the muscle fibres (Ramaswamy et al. 2011).

The accumulation of extracellular matrix particularly collagen, seems to be the result of incomplete repair of muscle following damage (Serrano and Munoz-Canoves 2010). Skeletal muscle regeneration following injury depends upon a series of well-co-ordinated events involving numerous cell types that modify the microenvironment of the damaged muscle which is essential for normal muscle regeneration to preserve muscle architecture. During ageing, this remodelling becomes dysregulated. Dysfunction in remodelling is coupled with a switch in myogenic progenitor cells from a myogenic to fibrotic fate (Shefer et al. 2006; Brack et al. 2007) or an adipogenic fate (Vettor et al. 2009; Pisani et al. 2010) suggesting satellite cells as a possible source of intramuscular fibrotic and fat deposition. This change in cell fate is possibly due to changes in the Wnt signalling pathway which has been shown to be involved in the myogenic fate of satellite cells and increased Wnt signalling has been shown to occur in ageing muscle (Vertino et al. 2005; Brack et al. 2007). Alternatively, changes in the inflammatory responses, such as those seen during ageing, may also play a role in determining cell fate (Wang et al. 2015).

Increases in collagen deposition lead to an increase in advanced glycation end products (AGE) in skeletal muscle in humans (Haus et al. 2007) and changes in total muscle collagen and the endomysium and perimysium have been shown to correlate with the increase in stiffness of muscle and a decline in muscle tension with age (Alnaqeeb et al. 1984). However, Goldspink et al. showed no difference in the transcription levels of collagen in the muscle of old mice (Goldspink et al. 1994). Given that total collagen levels are elevated in muscle of old mice these data suggest that there may be a reduction in collagen degradation possibly due to the increased collagen crosslinking, making the collagen somewhat resistant to degradation by collagenase.

## Changes in the neuromuscular system during sarcopenia

During ageing, a decrease in motor unit number in various muscles of humans (Piasecki et al. 2015) rodents (Ling et al. 2009; Sheth et al. 2018). A decrease in the number of motor axons innervating fibres has been observed in rodents (Ansved and Larsson 1990) and humans (Tomlinson and Irving 1977). Denervation leads to the sprouting of axons of existing functional nerves to innervate fibres in close proximity. This is known as motor unit remodelling and is evidenced by an increase in reinnervation is old mice (Larsson 1995). Reinnervation is speculated to cause some of the age-related fibre- type switching that occurs (Larsson et al. 1978; Andersen 2003; Lee et al. 2006), as slow motor neurons may be more adapted to reinnervation which leads to an age-related loss in fast motor neurons (Kadhiresan et al. 1996). If reinnervation does not occur, it is likely that the muscle fibre will eventually undergo cell death (Borisov and Carlson 2000; Borisov et al. 2001; Vasilaki et al. 2016). Research has provided evidence that neuromuscular remodelling is a pre-requisite for muscle atrophy (Deschenes et al. 2010; Sheth et al. 2018). Sheth et al. have shown that the decrease in motor unit number occurred before the loss of muscle function and the loss of motor unit connectivity seen during ageing correlates with muscle size and contractibility (Sheth et al. 2018). Deschenes et al. also showed that denervation precedes muscle loss in rats during ageing (Deschenes et al. 2010). However this is still poorly understood, due to the confines to study the neuromuscular system during ageing, one of these limitations is that it is not always possible to use human nerve and muscle tissues and these processes are only able to be studied using in vivo animal models or ex vivo co-cultures that require the use of spinal cord explants from animal embryos as a source of motor neurons. However, new developments in techniques to derive functional motor neurons from human pluripotent stems cells now allow development of alternative approaches.

## Increases in reactive oxygen species and alterations in antioxidant defence systems during sarcopenia

Reactive oxygen species (ROS) are extremely reactive molecules and have important roles in metabolism and cell signalling (Thannickal and Fanburg 2000). Though ROS have important functions in cells, when in excess if not eliminated by the antioxidant defence system, ROS can be damaging to cellular marcomolecules such as lipids, proteins and DNA, leading to cell death.

ROS are increased in the satellite cells of older subjects (Minet and Gaster 2012) which may contribute to the loss of regeneration potential in muscles of older animals and humans. The basal levels of ROS are also increased in mouse muscle during ageing (Palomero et al. 2013). This increase in ROS is thought to be detrimental to skeletal muscle as it is reflected by increases in markers of oxidative damage such as an increase in protein carbonyl and malonaldehyde and oxidation of lipids, DNA and proteins in the muscles of old mice (Mecocci et al. 1999; Broome et al. 2006; Sakellariou et al. 2016). This modified redox status has also been shown to be detrimental for other processes such as calcium transport (Fulle et al. 2003) and increased degradation of important proteins such as myogenic proteins, impaired autophagy (Scherz-Shouval et al. 2007) and inhibition of differentiation of muscle cells (Ardite et al. 2004; Sandiford et al. 2014).

ROS are eliminated by the antioxidant defence system. During ageing, it has been shown there is a constituent upregulation of the antioxidant defence system activity in skeletal muscle (Vasilaki et al. 2006; Palomero et al. 2013; Sullivan-Gunn and Lewandowski 2013). Following a stress such as muscle contraction, there is no further increase in antioxidant defence enzyme activities in the muscle of old humans and animals (Vasilaki et al. 2006; Ryan et al. 2008) potentially leaving the cells exposed to oxidative damage.

The contribution that ROS play in the muscle ageing process remains unclear. Interestingly, overexpression of copper/zinc (Cu/Zn) superoxide dismutase (CuZnSOD) leads to muscle atrophy (Rando et al. 1998) whereas the deletion of CuZnSOD results in the inability of muscle to adapt to stress and decreased muscle force generation in mice (Muller et al. 2006; Vasilaki et al. 2010; Larkin et al. 2011; Sakellariou et al. 2014b) suggesting that the redox balance is an important modulator of sarcopenia.

## Dysfunction of mitochondria during sarcopenia

Mitochondria are essential for providing the ATP required for muscle contraction and are also central to the redox regulation and quality control of the cell and therefore for the viability of muscle cells. Given this essential role of mitochondria in skeletal muscle maintenance and survival, alterations in mitochondria are considered one of the primary contributors driving the sarcopenic process.

The role of the mitochondria in sarcopenia was proposed by Miquel et al. in the mitochondrial free radical theory of ageing (Miquel et al. 1980). This stated that mitochondrial dysfunction in ageing occurs from the increase in ROS and blunted antioxidant defences; these damaging effects change the redox status of the cell which in turn leads to mutations in the mitochondrial DNA (mtDNA) leading to the production of dysfunctional components of the electron transport chain (ETC). Impairment of the ETC leads to compromised oxidative phosphorylation which causes a further rise in ROS, causing a vicious circle which exacerbates the ageing phenotype (Miquel et al. 1980).

This hypothesis was confirmed in skeletal muscle by studies showing that during ageing there was an increase in ROS, mtDNA deletions and mitochondrial dysfunction which were associated with skeletal muscle atrophy in non-human primates (Lee et al. 1998a) rodents (Wanagat et al. 2001) and humans (Bua et al. 2006). Interestingly, these observations were not seen in the phenotypical normal regions of the muscle fibres. Furthermore mice which contain error prone mtDNA polymerase accumulate high levels of mtDNA mutations and show severe muscle atrophy due to increased apoptosis (Kujoth et al. 2005). Further studies showed that mtDNA mutations lead to increased ROS production (Logan et al. 2014) and overexpression of antioxidants have been shown to protect against some of the oxidative damage as well as changes to mitochondrial respiration and ATP production in skeletal muscle (Lee et al. 2010) which prevent age related mitochondrial dysfunction. These data suggest that both ROS and mitochondrial dysfunction are likely to contribute to sarcopenia. However, more recently the mitochondrial free radical theory of ageing has become debatable as non-mitochondrial sources of ROS generation have been identified (Sakellariou et al. 2013, 2014a; Jackson and McArdle 2016).

Mitochondria in the muscle of sarcopenic individuals also show increased fusion and decreased fission (Yoon et al. 2006) and impairment of mitochondrial autophagic (Gouspillou et al. 2014) and proteasomal machinery (Marzetti et al. 2008). The release of damaged mitochondrial components into the extracellular matrix correlates with increases in pro-inflammatory cytokines in the plasma of elderly humans (Pinti et al. 2014). Mitochondria undergo complex morphological changes during aging which are also likely to affect their function (Leduc-Gaudet et al. 2015) and thus give further evidence for a role for dysfunctional mitochondria in sarcopenia.

## Increased inflammation during sarcopenia

The inflammatory response is the secretion of pro-inflammatory mediators in response to the appropriate stimuli such as toxins, bacteria, foreign bodies or infection and restores homeostasis and initiates repair. The acute pro-inflammatory state is vital for the repair of cells but too much for too long is thought to be detrimental; for example chronic low grade inflammation has been associated with ageing and has been implicated in numerous conditions and diseases (Lagrand et al. 1999; Duncan et al. 2003; Frischer et al. 2009).

Low level chronic inflammation coupled with immunosenescence, the decline in the function of the immune system with age, that occurs in ageing has been termed ‘inflamm-ageing’ (Franceschi et al. 2000). Inflamm-ageing has been associated with numerous age-related diseases and conditions (Chung et al. 2009) and has been implicated as a major contributor to sarcopenia (Schaap et al. 2006, 2009).

Serum levels of TNF-α, IL6 and C-reactive protein (CRP) are all increased in ageing and have been proposed to be important mediators of sarcopenia as changes are correlated with a decrease in muscle mass (Pedersen et al. 2003; Aleman et al. 2011; Bian et al. 2017), performance (Thalacker-Mercer et al. 2010), function (Bautmans et al. 2011), strength (Tiainen et al. 2010; Norman et al. 2014) and fitness (Levinger et al. 2010). As well as increasing myokine production (Lightfoot et al. 2015). It should be noted however that TNF-α, IL6 and CRP have all been shown to have beneficial effects in skeletal muscle growth; IL6 and TNF-α at low levels has been shown to cause satellite cell proliferation and differentiation (Li 2003; Kurosaka and Machida 2013), therefore it is likely that the effect of systemic inflammation on muscle mass and function during ageing may only occur when it surpasses a certain threshold and/or persists for an extended period (Degens 2010).

The increase in inflammation leads to a further increase in ROS production by skeletal muscle (Li et al. 1998). As well as an increase in skeletal muscle cell apoptosis (Phillips and Leeuwenburgh 2005), inflammation has also been proposed to play a role in the anabolic resistance described previously and high levels of inflammation have been associated with catabolism of skeletal muscle (Li et al. 1998; Cuthbertson et al. 2005).

## Current therapies for sarcopenia

The ageing population is growing substantially with 617 million people worldwide currently 65 and over and demographic analysis predict that this will increase to 1.6 billion by 2050 (He et al. 2016). Although there have been improvements in lifespan, the same advances have not been made in health-span, meaning that the extra years of living are under poor health. Sarcopenia is a major contributor to frailty in the older population resulting in further immobility with a loss of independence, as well as increasing the risk of other chronic diseases and morbidity (Coin et al. 2013). This immobility and co-morbidities can be further escalated through lifestyle choices such as a sedentary diet and a poor diet. Thus sarcopenia has major socio-economic costs—in 2000 the US spent 1.5% of their national budget ($18.5 billion) on sarcopenia (Janssen et al. 2004), signifying the importance of finding a treatment or preventative therapy for sarcopenia. This section focuses on modifiable lifestyle factors which are economically resourceful compared with drug interventions.

## Physical exercise and exercise

The importance of physical activity in preventing sarcopenia has been shown in studies where people who are less physically active have a higher chance of developing sarcopenia (Lee et al. 2007). It is generally thought that exercise and physical activity are beneficial and can attenuate some of the detrimental effects of unloading and bed rest on muscle loss in adult and old individuals (Caiozzo et al. 2009; Belavy et al. 2014; McMahon et al. 2014; Valenzuela et al. 2018). It is important to define the differences between exercise and physical activity. Physical activity is defined as bodily movements that are produced by skeletal muscles and result in energy expenditure; examples of this include walking and house chores. Exercise is a subset of physical activity that is planned, structured, and repetitive and has as a final objective to improve or maintain physical fitness (Caspersen et al. 1985). Lifelong exercise was shown to be associated with modest improvements in muscle mass in the quadriceps of mice (McMahon et al. 2014) and lifelong triathlon training is able to preserve muscle mass in the mid-thigh of humans (Wroblewski et al. 2011). However, the benefits of exercise on muscle function are controversial since lifelong exercise did not prevent the loss of strength when it was shown that master athletes still undergo a loss in strength, power and endurance with age (Grassi et al. 1991; Kayani et al. 2008). Others have also shown no correlation between physical activity and the maintenance of muscle mass (Mitchell et al. 2003) and only a higher level of physical activity and not ‘leisure-time’ activity are able to prevent or delay some sarcopenic effects (Raguso et al. 2006).

There are various different exercise programmes that are recommended for the older population in an attempt to combat sarcopenia.

## Resistance training

Resistance training is the requirement to generate force to move or resist weight such as weight lifting/push ups/leg press. Numerous studies have shown beneficial effects of resistance training in the function of skeletal muscle of older people; increasing muscle mass and strength (Fiatarone et al. 1994; Maltais et al. 2015; Tsuzuku et al. 2018) as well as cross-sectional area of myofibres (Fiatarone et al. 1994; Leenders et al. 2013; Ribeiro et al. 2017) and motility (Fiatarone et al. 1994; Liu and Latham 2009).

Improvements in muscle function following resistance training are thought to be due to an improved neuromuscular system (Taaffe et al. 1999), increased protein synthesis and attenuation of anabolic resistance (Schulte and Yarasheski 2001) and this is associated with an increase in the satellite cell content of type II fibres (Verdijk et al. 2009a; Leenders et al. 2013). Resistance training has also been linked with a decrease in catabolic and increase in anabolic pathways (Ribeiro et al. 2017).

## Aerobic training

Aerobic training stimulates the heart and blood flow and provides cardiovascular conditioning such as running, cycling and swimming. Aerobic exercise has been shown to result in increased cross-sectional area of muscle fibres and hypertrophy of muscles of older humans (Schwartz et al. 1991; Konopka et al. 2013). However, the effects of aerobic exercise are not as well established as resistance exercise and it is likely that the hypertrophic effects of aerobic exercise depend on the frequency, intensity and length of exercise.

The effects of aerobic exercise on skeletal muscle are primarily through increases in mitochondrial proteins such as cytochrome C and PGC-1α (Short et al. 2003; Konopka et al. 2013). Increases in mitochondrial biogenesis result in improved mitochondrial function, metabolic control and respiratory capacity (Coggan et al. 1992; Short et al. 2003) consequently increasing the endurance of the individual. Furthermore, long term aerobic exercise programmes have shown an ability to reduce ROS production by muscle in old people (Ghosh et al. 2011). Aerobic exercise has also been shown to decrease anabolic resistance through the upregulation of protein synthesis through the Akt/mTOR pathway (Fujita et al. 2007), as well decreasing inflammation (Kohut et al. 2006).

## Other forms of physical activity

Other forms of physical activity include power training. Power declines at a rate of 3–4% per year in older people and this is detrimental for everyday activities such as climbing stairs. To improve power, fast shortening resistance training is implemented. Improvements in skeletal muscle power (Fielding et al. 2002; Henwood and Taaffe 2005; Reid et al. 2008) and in the ability to carry out every day activities in older people have been seen following power exercise regimes (Henwood and Taaffe 2005). These improvements are thought to be due to changes in the neuromuscular junction that allow better recruitment of the motor units and therefore an increase in the firing rate of fast twitch fibres (Fielding et al. 2002; Reid et al. 2008). Reid et al. have shown power training to be more effective than slow velocity resistance training (Reid et al. 2008).

Many suggested physical activities may be too intense for older adults to maintain over a prolonged time. To combat this, less impact exercises such as whole-body vibrations and whole body electro-myostimulation have been developed. These techniques use impulses that cause involuntary contractions of the muscles to preferentially recruit the fast twitch fibres that are most affected by ageing and this approach has been shown to increase maximum isometric strength and muscle mass (Kemmler et al. 2010, 2014) and grip strength in older women (Stengel et al. 2015).

## Protein intake and calorie restriction

Protein and other nutrients are vital for the protein synthesis required for muscle growth and maintenance. Therefore it is proposed that nutritional intake may play a role in sarcopenia and altering nutritional intake may be able to relieve some symptoms of sarcopenia.

## Increase in protein intake

In addition to the anabolic resistance that occurs with age, around 30–40% of women and 20–40% of men over 50 do not reach the recommended daily intake of protein and it is has been shown that a low protein diet can be detrimental to muscle (Oumi et al. 2000; Balasa et al. 2011; Tarry-Adkins et al. 2016). Therefore, a considerable number of studies examining interventions against sarcopenia have focused on increasing protein intake.

Studies have shown that increasing the overall amount of protein intake can at least overcome the anabolic resistance in older people leading to an increase in protein synthesis, muscle mass and decreased proteolysis in rodents (Mosoni et al. 2014) and humans (Genaro et al. 2015; Moore et al. 2015; Norton et al. 2015; Verreijen et al. 2015).

The importance of the essential amino acid profile, digestibility and bioavailability of ingested protein on the anabolic potential of protein was demonstrated in studies where anabolic resistance was overcome by increasing the percentage of leucine or essential amino acids contained in the ingested protein rather than the total amount of protein (Volpi et al. 2003). Increased protein synthesis was also achieved by inhibiting co-ingestion of carbohydrates and protein (Katsanos et al. 2006). This improvement in anabolic resistance in the old is likely to be through the upregulation of the Akt/mTOR pathway as well as decreasing proteolysis and autophagy (Volpi et al. 2003). Increases in Akt/mTOR pathway and decreases in proteolysis have also been shown in vitro (Sato et al. 2014) and in vivo resulting in increased muscle mass when used as a single supplement of leucine or in combination with other nutrients (Sato et al. 2013, 2015). Leucine supplementation led to improved muscle regeneration in old rats through a decrease in inflammation and an increase in satellite cell proliferation resulting in an increase in the cross-sectional area of regenerated fibres compared with control animals (Pereira et al. 2015).

In contrast, meta-analysis of protein supplementation studies (Xu et al. 2014) showed no difference between the effect of protein supplementation and that of a placebo groups on muscle mass, protein synthesis and muscle strength in older men (Dirks et al. 2014) or women (Zhu et al. 2015). Furthermore, (Russ et al. 2015a, b) showed that protein supplementation attenuated muscle degradation through decreasing Murf1 expression, this did not translate to any functional benefits to muscles of old rats (Russ et al. 2015b). However these discrepancies may be explained by the times at which intake of protein occurred; Symons et al. (2007) showed that a 90 g of protein meal in humans does not cause more protein synthesis than a 30 g meal in humans (Symons et al. 2007). This suggests that ingestion of more than 30 g of protein in one meal is an energetically inefficient means of protein synthesis and that protein intake should be spread out throughout the day to optimise muscle protein synthesis.

Despite the benefits of increased protein synthesis on sarcopenia, it is important to note that high protein diets (3 g protein × kg fat-free mass (FFM)(− 1) × day(− 1) have been linked to a decrease in the glomerular filtration rate in older people, suggesting that high levels of protein may have damaging effects on the kidney (Walrand et al. 2008) and undesirable effects on the musculoskeletal system were seen when high protein diets led to negative a calcium balance that could lead to osteoporosis in men (Allen et al. 1979). Thus prescribing increased intake of protein in old people is controversial.

## Calorie restriction

Calorie restriction is thought to be one of the most effective interventions of attenuating ageing. Restriction of the number of calories eaten has been proved to be life-extending in numerous species (Weindruch et al. 1986; Lakowski and Hekimi 1989; Jiang et al. 2000) as well as reducing all-cause mortality in rhesus monkeys (Colman et al. 2014).

The benefits of calorie restriction have been extended into sarcopenia. In rats, a 6 week 20% reduction in calorie intake led to an attenuation of age-related loss of muscle mass and function in the soleus and gastrocnemius muscles through an upregulation of PGC-1α (Joseph et al. 2013a). Calorie restriction also preserved fibre number and type and protected mitochondrial DNA from deletion (Lee et al. 1998b). In rats, calorie restriction decreased apoptosis and protected from oxidative stress (Dirks and Leeuwenburgh 2004) as well as a decrease in the overall oxidation status in skeletal muscle (Hepple et al. 2008). These data suggest that calorie restriction prevents sarcopenia potentially through an inhibition of apoptosis and enhancement of the mitochondrial function and this has been shown to occur through the upregulation of the NAD-deacetylase Sirt1 (Cohen et al. 2004). Sarcopenia was also attenuated by calorie restriction in the rhesus monkey (Colman et al. 2008).

The relevance and beneficial effects of human calorie restriction is shown in studies which have shown positive effects in diseases such as diabetes and atherosclerosis (Fontana et al. 2004; Weiss et al. 2006). Importantly Mercken et al. showed a long term 30% reduction in calorie intake in humans changed the transcriptional profile in skeletal muscle of an older individual similar to that of a younger subject, increased the production of antioxidants and decreased inflammation (Mercken et al. 2013). This suggests that the benefits of calorie restriction can be extended into human muscle however a lot more work is needed in this area. It is likely that for a high adherence and for beneficial effects of a calorie restricted diet, this would have to be implemented at a younger age and it would be vital for people to be well informed about calorie intake. This would also need to be looked at on an individual basis as insufficient nutrition is already a problem for a lot of elderly people therefore, if misinformed it could lead to the malnutrition of patients which has been shown to result in a lower muscle mass (Pierik et al. 2017).

## Protein supplementation paired with exercise

Given the benefit of exercise and protein intake on sarcopenia, numerous studies have shown that combined together, protein and exercise can increase muscle strength and mass in the old (Tieland et al. 2012; Shahar et al. 2013; Maltais et al. 2015; Palop et al. 2015).

The ability of protein supplementation to increase muscle mass and strength further than with exercise alone is debateable. Some research groups have suggested that protein supplementation will only enhance exercise-induced muscular improvements if there is an existing protein deficiency (Verdijk et al. 2009b). This is particularly relevant in those who do not already reach the recommended daily intake of protein, where the amount and distribution of protein throughout the day alongside an effective exercise plan may play an essential part in whether the supplement will be effective.

## Future direction

Despite some evidence for the benefits of exercise and nutritional interventions on sarcopenia there is still no intervention that there is an agreement that is beneficial to sarcopenia. Studies looking at more pharmacological agents have been more promising. For example the inhibition of myostatin, a negative regulator of muscle mass, increased muscle size in mice and cattle (Lee 2007) and the use of sex hormones have improved muscle strength and mass (Stárka 2006). More recently microRNAs, small RNAs that post transcriptionally regulate gene expression, have been shown to be involved in skeletal muscle development (Goljanek-Whysall et al. 2012) and the levels of miRNAs dysregulated in ageing humans (Drummond et al. 2011) and mice (Soriano-Arroquia et al. 2016a, b). Furthermore the restoration of level of miRNAs have led to an improved muscle phenotype in old mice, but also the imitation of ageing miRNA levels in younger mice have also resulted in detrimental effects in the muscle (Soriano-Arroquia et al. 2016a). Furthermore, miRNAs have also been shown to be involved in the adaption of skeletal muscle following exercise (Russell et al. 2013) thus showing the potential for miRNAs paired with a personalised exercise regime as a treatment for sarcopenia.

## Conclusion

Skeletal muscle is a vital organ to the body and the age-related changes that occur in the muscle are detrimental to the correct functioning of skeletal muscle and leads to the loss of independence. The ever-increasing ageing population is an important socio-economic problem. The changes that occur in sarcopenia have been described in the sections above and there is a vast amount of evidence that these changes contribute to the resulting phenotype in skeletal muscle wasting.

Despite the huge amount of work looking at life-style changes on sarcopenia, whether these changes can prevent or cure sarcopenia is still to be established. The contrast in the results from these studies suggests that the responsiveness of individuals to exercise and changes in nutritional intake may depend on the individual and stage of sarcopenia that is occurring. It may suggest that complete personalised regimes, maybe in conjunction with pharmacological interventions are required for full function of the muscle during later life.
